# Tumour immune escape via P2X7 receptor signalling

**DOI:** 10.3389/fimmu.2023.1287310

**Published:** 2023-10-30

**Authors:** Ricardo M. Sainz, Jorge Humberto Rodriguez-Quintero, Maria Constanza Maldifassi, Brendon M. Stiles, Erik Wennerberg

**Affiliations:** ^1^ Division of Radiotherapy and Imaging, The Institute of Cancer Research, London, United Kingdom; ^2^ Department of Cardiovascular and Thoracic Surgery, Albert Einstein College of Medicine, Montefiore Health System, Bronx, NY, United States

**Keywords:** P2X7 receptor, ART1, CD38, CD39, tumour microenvironment, tumour immune escape, cancer immunotherapy

## Abstract

While P2X7 receptor expression on tumour cells has been characterized as a promotor of cancer growth and metastasis, its expression by the host immune system is central for orchestration of both innate and adaptive immune responses against cancer. The role of P2X7R in anti-tumour immunity is complex and preclinical studies have described opposing roles of the P2X7R in regulating immune responses against tumours. Therefore, few P2X7R modulators have reached clinical testing in cancer patients. Here, we review the prognostic value of P2X7R in cancer, how P2X7R have been targeted to date in tumour models, and we discuss four aspects of how tumours skew immune responses to promote immune escape via the P2X7R; non-pore functional P2X7Rs, mono-ADP-ribosyltransferases, ectonucleotidases, and immunoregulatory cells. Lastly, we discuss alternative approaches to offset tumour immune escape via P2X7R to enhance immunotherapeutic strategies in cancer patients.

## Introduction

P2X receptors (P2XR) belong to the ligand-gated ion channel family of receptors (1) and are characteristically gated by extracellular adenosine triphosphate (ATP). In mammals, seven subunits are expressed (P2X1-7), and form homo-trimeric or hetero-trimeric structures with diverse pharmacological characteristics (2). Agonist binding sites are found between subunit interfaces consisting of mostly positively charged amino acids, where activation of the receptor induces the opening of a cation permeable channel (2). Among the P2X receptors, the P2X7R is the least sensitive to ATP with EC_50_ values in the mM range and is also the most slowly desensitizing (3). Here, binding of two ATP molecules causes the opening of the channel, with the subsequent permeation of Ca^2+^, Na^+^, and K^+^ ions, whereas the binding of the third ATP generates a dilation of the channel which is known also as the “macropore”, resulting in ATP-induced cell death (AICD) (3–6). Although previously well described in various physiological and pathophysiological states (7), recent interest in the P2X7R has risen because of its involvement in diverse inflammatory conditions, including cancer (8).

The P2X7R is widely expressed by cells of the immune system. Because of the high threshold of ATP needed to activate the receptor, which can be found in conditions of cellular damage, it is considered a sensor for immunogenic danger signalling or a damage-associated molecular pattern (DAMP) which can trigger recruitment of various activated immune cells to the site of tissue damage, or alternatively AICD depending on ATP concentration (9–13). However, ATP can also be released through regulated mechanisms and in this manner control diverse immune-cell functions in an autocrine/paracrine fashion. In fact, activation of T-cells induces ATP release through pannexin-1 (Panx1) channels stimulating in an autocrine manner P2X7R, prompting cytokine release and proliferation (14). Furthermore, P2X7R plays a role in T cell migration and egress from lymph nodes following activation and differentiation by promoting shedding of L-selectin and by paracrine regulation of T cell motility via the P2X7R (15–17).

Dendritic cells (DCs) respond to ATP stimuli via P2X7R by activating Panx1 and causing an autocrine signalling loop that results in maturation of DCs as well as promoting cellular migration to lymph nodes facilitated by upregulation of the chemokine receptors CCR7 and CXCR4 (18, 19). P2X7R-induced upregulation of co-stimulatory molecules through a nuclear factor kappa B (NF-kB)-dependent mechanism promotes helper T (Th) cell differentiation (20, 21). Other innate immune populations show a similar effect, including monocytes (22), macrophages (23), eosinophils and neutrophils, facilitating actin polymerisation for transendothelial migration (24, 25). Further, P2X7R activation of DCs and other myeloid cells can trigger NLRP3 inflammasome complex induction, associated with efflux of cellular potassium, influx of calcium, reactive oxygen species (ROS) generation, and mitochondria depolarization. Altogether, it leads to the release of the pro-immunogenic cytokines interleukin (IL)-1β and IL-18, eventually inducing pyroptosis, a highly immunogenic form of cell death which further propagates the inflammatory signal (26, 27).

The P2X7R influences cell plasticity, differentiation, and metabolic fitness of immune populations. Genetic ablation of the P2X7R in the LCMV Armstrong infection model showed a normal expansion of effector T cells. However, the central memory T cell (T_CM_) and tissue-resident memory T cell (T_RM_) subsets were affected and lacked long-lasting protection against future reinfections. This was suggested to be due in part to metabolic impairment, specifically, a lower mitochondrial mass and a compromised spare respiratory capacity (28, 29). On the other hand, reduced mitochondrial mass makes T cells from P2X7R-deficient mice less susceptible to cell senescence derived from P2X7 activation (30).

## P2X7R in tumour immune escape

P2X7R is expressed in most solid and haematological cancers, and there is ample evidence that tumour cell expression of P2X7R promotes proliferation, metabolism and facilitates tumour invasion and metastatic dissemination (31–35). While P2X7R expression has also been proven essential for mounting effective anti-tumour responses, preclinical studies evaluating how P2X7R governs anti-tumour immune responses have shown widely different and sometimes contradicting outcomes (36, 37). Ghiringelli and colleagues showed that P2X7R expression is mandatory for DC-mediated sensing of immunogenic cell death following anti-cancer therapy (36). Adinolfi and colleagues confirmed the derogatory immune effect of P2X7R-deletion in tumour-bearing mice, in particular the reduced activity of DCs (37). Further, a study by De Marchi and colleagues revealed that genetic deletion of P2X7R had adverse effects on tumour-infiltrating T cells and promoted tumour growth while systemic P2X7R antagonism in the same tumour model benefited T effector cells and reduced tumour burden (38).

In the context of adoptive T cell therapy, two studies assessing how P2X7R-expression affects the performance and anti-tumour effectiveness of transferred CD8 T cells reached opposite conclusions. Romagnani et al. described that P2X7R-expressing tumour-infiltrating lymphocytes (TILs) display signs of cellular senescence while Wanhainen et al. showed that they have superior mitochondrial fitness, proliferation and apoptosis-resistance compared to P2RX7-deficient TILs (30, 39). A potential explanation for the discrepancy between the two studies was the use of different T cell culture conditions prior to infusion. Work from Koch-Nolte’s lab suggest that discrepancies in immune readouts from pre-clinical P2X7R studies may be explained by selection bias following NICD- and AICD-mediated elimination of P2X7R-expressing immune cells exposed to high concentrations of NAD^+^ and ATP respectively during tissue dissociation and cell preparation (40, 41).

**Figure 1:**
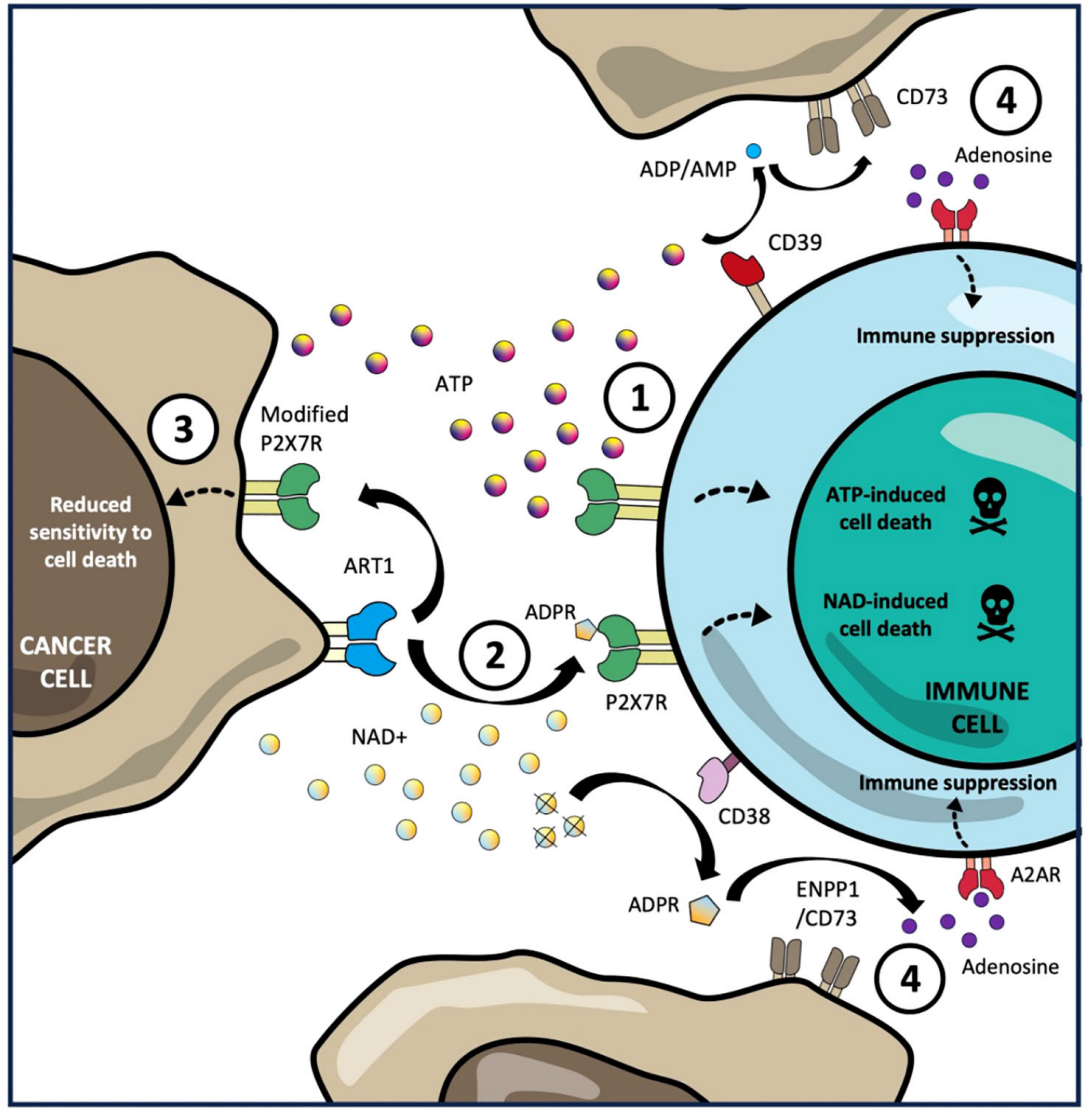
Tumour immune escape mechanisms through P2X7R signalling. 1. Sustained exposure of high concentrations of extracellular ATP, produced in the tumour microenvironment (TME), is sensed by the P2X7R on tumour infiltrating lymphocytes triggering macropore formation ATP-induced cell death (AICD). 2. Expression of ART1 by tumour cells can utilize extracellular NAD+ in TME mono-ADP-ribosylate the P2X7R on TILs in trans resulting in NAD-induced cell death (NICD). 3. Expression by tumour cells of non-pore functional variants of the P2X7R with low affinity for AICD and NICD allows them to avoid cell death while still exploiting P2X7R-mediated proliferation and growth signalling. 4. CD39-mediated catabolism of extracellular ATP and CD38-mediated catabolism of extracellular NAD into AMP and ADPR respectively, is converted into immunosuppressive adenosine by CD73, which is frequently overexpressed by tumour cells, resulting in polarization of intratumoural immune cells towards a regulatory phenotype (including Tregs and macrophages) as well as reduced proliferation, cytokine production and cytotoxic function of effector lymphocytes.

While the picture of how P2X7R regulates anti-tumour immunity is complex, there are key emerging areas where tumours have been shown to skew P2X7R signalling to promote immune escape. These are summarized below and illustrated in **Figure 1**.

## Non-pore functional P2X7R (nfP2X7R) variants

The high concentration of extracellular ATP found in tumours is proposed to drive the expression of modified P2X7Rs in cancers to favour tumorigenesis and progression (42, 43). These include single nucleotide polymorphisms and splice variants, leading to loss of the P2X7R macropore function, as well as reduced sensitivity to activation by MARylation which has further been associated with tumour growth and immune escape (**Figure 1**) (44, 45). In 2019, Gilbert et al, showed that the nfP2X7R E200, which does not induce large pore formation and cell death upon ATP-binding was expressed broadly across different human cancers. The authors showed that exposure of tumour cells to high concentrations of ATP expression of E200 nfP2X7R which was essential for survival of the cancer cells (43).

Antibodies targeting E200 nfP2X7R, was tested in an open-label phase I non-randomized clinical trial as a topical agent (BIL010t) for the treatment of basal cell carcinoma of the skin. The trial demonstrated that after being applied to primary lesions twice a day for 28 days (n=21), 65% of patients underwent reduction of lesion size, while 20% and 15% showed no changes, and increase in size, respectively. Although complete pathologic response was only observed in 3 of the patients after excision, treatment compliance was high, and the treatment was well tolerated (46). Further, A recent paper has shown that chimeric antigen receptor (CAR)-T cells targeting nfP2X7Rs have potent cytotoxic potential against human cancer cells. When adoptively transferred to NOD-scid-IL2Rγ^null^ (NSG) mice, CAR-T cells were able to traffic to and infiltrate orthotopic breast and prostate tumours (47).

## Mono-ADP-ribosyltransferases (ARTs)

In the presence of ARTs, the P2X7R can be activated by nicotinamide adenine dinucleotide (NAD^+^), a nucleotide that can be released extracellularly after cellular damage or from activated T cells (48, 49). In mice, expression of ART2 by peripheral T cells, allows them to mono-ADP-ribosylate (MARylate) the P2X7R in cis (50). This ART2-mediated MARylation results in Ca^2+^ increase, exposure of the phospholipid phosphatidylserine (PS), formation of a macropore, and ultimately NAD-induced cell death (NICD) (49–52). Importantly, the NAD^+^ concentration threshold for NICD via MARylation of the P2X7R is significantly lower than for P2X7R-mediated AICD and this process is irreversible (52). Stark and colleagues showed, in mouse models of tissue damage and infection, that ART2-mediated NICD of T cells serves as a mechanism for enrichment of antigen-specific T cells in inflamed tissues.

ART2 is a pseudogene in humans and no T cell-expressed ART with similar immune homeostatic function has been identified to date. However, ART1, which is expressed in various human tissues including lung epithelium and skeletal muscle as well as on a subset of CD39^+^ CD4 T cells, has been shown to MARylate immune cells in trans (53, 54). In non-small cell lung cancer (NSCLC), ART1 expressed by tumour cells has been characterized as a novel pathway of immune escape. Wennerberg and Mukherjee et al. showed that ART1 expression was associated with reduced tumour infiltration of P2X7^+^ CD8 T cells in non-small cell lung cancer (NSCLC) patients, while in murine immune competent lung tumour models, ART1 knockdown decreased tumour growth (55). Correspondingly, ART1 blockade with a therapeutic monoclonal antibody (22C12) reduced the growth and dissemination of ART1 expressing tumours in mice and promoted tumour infiltration of activated P2X7R^+^ CD8 T cells (**Figure 1**) (55).

## Ectonucleotidases

Through its catabolism of eATP, CD39 produces precursors for CD73, which is overexpressed in several cancers, to generate immunosuppressive adenosine in the tumour microenvironment (TME). This has prompted testing of CD39 inhibitors in preclinical mouse models, which have shown impressive T cell and NK cell-mediated anti-tumour effects in immunogenic mouse tumour models (56–58). While CD39 expression on T cells has traditionally been described a marker of exhaustion and dysfunctionality, emerging patient reports show that CD39-expressing T cells are enriched in solid tumours, where they are shown to be preferentially tumour antigen-specific (59–61). Exposure of T cells and NK cells to high concentrations of ATP result in AICD and reduced cytotoxicity respectively in P2X7R-dependent manners (62, 63). Presumably, modulation of CD39 expression following ATP exposure, serves as a cytoprotective function for lymphocytes to maintain crucial effector functions in the TME (**Figure 1**) (38).

CD38 is primarily expressed on activated T cells and through its generation of cADPR, CD38 can function as a second messenger for Ca^+^ mobilization regulating T cell activation (64, 65). Further, CD38 expression clusters in the immune synapse upon T cell receptor (TCR) interaction with antigen-presenting cells, suggesting that CD38 plays a role in regulating T cell function (66). While CD38 expressed on tumour cells can mediate immune resistance by providing precursors for adenosine generation via the non-canonical pathway mediated by ENPP1 (CD203a) and CD73 (67, 68), its NADase function in immune cells is important for protection against ART-mediated NICD under NAD-rich conditions (**Figure 1**). Indeed, Krebs et al. showed that ART2-mediated mono-ADP-ribosylation following eNAD exposure was elevated in T cells lacking CD38 expression while Adriouch et al. showed that CD38-deficient mice experienced significantly more depletion of P2X7R^+^ T cells following NAD^+^ injection compared to wild-type mice (69, 70). In the tumour context, we have demonstrated that NICD of P2X7R^+^ CD8 T cells following exposure to recombinant ART1 is exacerbated in the presence of CD38-blocking antibodies. Consistent with these findings, analysis of P2X7R^+^ TILs from ART1-expressing human lung tumours showed enriched expression of CD38 (55).

## Immunoregulatory cells

Regulatory T cells (Tregs) are recruited and polarized by tumours to blunt anti-tumour immune responses (71). Tregs are not affected by physiological concentrations of ATP, whereas concentrations approaching 1 mM triggers Treg-mediated immunosuppression (72). In lymph nodes, the expression of P2X7R in CD4 naïve cells or in Treg cells, can induce a posterior polarization towards the Th1/Th17 phenotype (73). Also, Tregs show cell plasticity based on the context. For example, in the presence of IL-6 and ATP, they become Th17 cells (74). If Th17 cells co-express CD39, they can differentiate into the IL-10-producing Tr1 phenotype (75). In mouse tumour models, P2X7R-deficient mice have elevated intratumoural Tregs compared to wild type mice (38, 74, 76). In these mice, the cytokine profile is shifted from pro-inflammatory to immunosuppressive mediators including TGF-β (38). In leukaemia patients, treatment with the chemotherapy agent daunorubicin promoted Tregs through P2X7R-dependent polarization of tolerogenic DCs (77).

Further, P2X7R is expressed on macrophages and on myeloid-derived suppressor cells (MDSCs). In a Lewis Lung carcinoma model, P2X7R expression on tumour-associated macrophages (TAMs) favoured immunosuppressive M2 polarization and anti-programmed cell death protein-1 (PD-1) resistance was overcome by administration of P2X7R inhibitors (78). In a murine neuroblastoma model, P2X7R signalling by MDSCs was associated with increased suppressive function including production of TGF-β, Arginase-1, and reactive oxygen species (**Figure 1**) (79). Recent work suggests that Toll-like receptor (TLR) mediated activation of diverse types of immune cell subsets can be used as a strategy to activate an immune response in the TME (80). In this regard, as the P2X7R is known to enhance the release of pro-inflammatory cytokines in macrophages and DCs after TLR2 and TLR4 activation, a dual therapeutic strategy could be conceived to obtain an increased anti-tumour immune effect (13, 81, 82).

As in other immune cells, microglial activation of the P2X7R causes a polarization of these cells towards a pro-inflammatory state, and as such the receptor has a clear role in diverse neuroinflammatory diseases such as Parkinsons and Multiple Sclerosis (83, 84). Mice microglia is known to express the P2X7a variant, that although it shows no variation of ATP sensitivity, it renders it insensitive to ART2-mediated NICD (85). Meanwhile, through diverse mutational studies, it is known that the retention of pore formation capabilities seems to be important for P2X7R driven microglial activation, proliferation, and cytokine release (86).

## Prognostic value of P2X7R expression in cancer

In Acute Myeloid Leukemia (AML), P2X7R splice variants A and B have shown promising prognostic potential to identify patients with relapsing disease. P2X7RB has also been identified as a poor-prognosis marker in osteosarcoma, neuroblastoma, and lung adenocarcinoma (87, 88). In colorectal cancer, P2X7R expression is higher in undifferentiated tumours and has been associated with adverse oncologic features including invasiveness, advanced stages, metastatic disease, and worse overall survival (89). In addition, P2X7R-high tumours may correlate with increased carcinoembryonic antigen (CEA) expression, a tumour marker used for monitoring metastatic disease (90). In gastric cancer, P2X7R has also shown promise as a prognostic marker. In a study of gastric cancer specimens, P2X7R was overexpressed in specimens from patients with lymph-node metastases, vascular invasion, and advanced stages. Additionally, an inverse correlation was noted between P2X7R and CD8^+^ TILs (89).

In contrast, in hepatocellular carcinoma, intratumoral P2X7R expression did not correlate with oncologic outcomes. However, peri-tumoral expression of the receptor was inversely associated with overall survival in both an experimental and a validation cohort (91). Similarly, P2X7R expression has been correlated with decreased overall survival, and metastatic disease in metastatic melanoma. In this setting, splicing variants A and B have been associated with malignant transformation (88). High P2X7R expression was correlated with decreased overall survival in a cohort of patients with lung adenocarcinoma from The Cancer Genome Atlas (TCGA) dataset (92). In muscle-invasive bladder cancer, P2X7R has been reported as a negative predictor of overall survival (93). These findings have been validated through TCGA analysis (94). In the setting of renal-cell carcinoma, a study showed that P2X7R expression is an adverse prognostic indicator for postoperative cancer-specific survival (95).

Contrary to the above, a study showed that in non-small cell lung cancer, overexpression of P2X7R was associated with improved overall survival (96). However other studies have shown results that conflict with these findings (97). Similarly, a study showed that decreased P2X7R expression associated with development with cervical cancer in patients with epithelial precancerous lesions (98). Overall, most studies with clinical correlation associate high P2X7R expression with adverse prognosis and decreased survival in cancer. It is critical to note that most of the cited studies did not distinguish between tumour cell expression and immune cell expression of P2X7R or distinguish between distinct P2X7R isotypes, analyses that could potentially better stratify patients in terms of prognosis.

## P2X7R antagonists and agonists in preclinical models

In the preclinical setting, a variety of antagonists of P2X7R have been described in recent literature, including Brilliant Blue G (BBG), oxidized ATP (oxATP), KN-04, KN-62, A740003, and A438079, although all are not specific for P2X7 and may block other purinergic receptors (99, 100). In AML, P2X7R blockade with AZ10606120 resulted in reduced leukemic growth when co-administered with daunorubicin, a process mediated through the blockade of P2X7 splice variant B (87). In neuro-oncological malignancies, AZ10606120 was shown to inhibit growth of human glioblastoma cells (101) and systemic administration of AZ10606120 in nude/nude mice reduced ACN-derived tumor growth of neuroblastoma. This was associated with downregulation of the Akt/hypoxia-inducable factor 1-alpha (HIF-1a) axis, and reduced VEGF and vessel formation as well as reduced the expression of MYCN, a crucial oncogene in neuroblastoma (32). In pancreatic cancer, AZ10606120 a non-selective inhibitor of P2X7R, inhibited the growth of stellate cells, a promoter of pancreatic adenocarcinoma progression (102). In contrast, a study by Mohammed et al, the inhibitors A438079 and AZ10606120 showed no chemopreventive effect, but instead promoted progression of intraepithelial lesions to cancer (103). In murine colorectal cancer models, P2X7R blockade with A438079 and AZD9056, inhibited tumour cell invasion, migration and TGF-B1 induced metastases (104). In addition, in murine models (CT26-mP2X7R), P2X7R blockade with intratumoral oxATP injections lead to reduction in tumour size and growth (31). Lastly, bilirubin has been found to interact with P2X7R, and decrease phosphorylation of mammalian target of rapamycin (mTOR), signal transducer and activator of transcription 3 (STAT3), and glycogen synthase kinase-3 beta (GSK-3beta), thus reducing oncogenicity (105).

In pancreatic cancer *in-vivo* murine models, the anti-P2X7 agent KN-62 abrogated tumor proliferation promoted by ATP (106). Alternatively, in a breast cancer *in-vitro* model, KN-62 inhibited ion currents, ethidium uptake, and calcium uptake, suggesting appropriate anti-P2X7 function (34). A similar isoquinoline derivative, KN-04 (an inactive analog), was found to inhibit ion fluxes in the nanomolar range. Subsequently, it was shown that both KN-62 and KN-04 only partially block pore formation (107). *In vitro*, A740003 a P2X7R blocker, has shown to reduce primary melanoma growth and to activate anti-tumor immune responses (38). In addition, A740003 has demonstrated a reduction in melanoma spread and tumour dissemination *in-vivo* (88). P2X7R antagonists have also been useful in establishing the mechanistic effects of P2X7R in lung models. For example, a specific P2X7R inhibitor GSK1370319A, was used to demonstrate that the macropore function of P2X7R may be impaired in immune cells of lung adenocarcinoma (92). Adamantane-1-carbonyl thiourea derivatives have also been shown to inhibit P2X7R activity, especially P2X7RB (108). Several natural compounds have been shown to antagonize P2X7 and have therapeutic potential. Teniposide, a podophyllotoxin derivative, acts as a topoisomerase inhibitor and is used in several types of cancer (109). Emodin, an anthraquinone derivative, specifically inhibited P2X7R-mediated currents and was shown to block cancer invasiveness *in vitro* and in an *in-vivo* zebrafish model of micrometastases (110). The agent GSK-1482160, a P2X7 blocker designed to target inflammatory conditions, was tested in a first-in-human blinded placebo-controlled study that was completed in 2009. The results of this study supported that the compound reduces the efficacy of ATP at the P2X7 receptor without affecting its affinity. With 29 subjects included, no safety or tolerability concerns were identified except for one case of asymptomatic accelerated idioventricular rhythm at the top dose (111).

Extracellular ATP has also been shown potential as anticancer therapy. In a study using a human prostate xenografts intraperitoneal injection of extracellular ATP resulted in significant tumour regression (112). However, this strategy failed to show clinical efficacy in a phase II study (113). In osteosarcoma, the P2X7R agonist benzoyl ATP (BzATP) promoted tumour proliferation and spread of osteosarcoma throughout the bone matrix. BzATP has also shown to have anti-tumour effect in glioblastoma stem cells (114). Non-nucleotide P2X7R agonists have also shown anti-tumour activity in pre-clinical models. A positive allosteric modulator against P2X7R, HEI3090, induced immune-mediated tumour regression in combination with anti-PD-1 antibodies in the immunotherapy-resistant Lewis Lung Carcinoma model (115),

## Future strategies

It has become clear that P2X7R is essential for innate immune cell sensing of immunogenic cell death, which plays an important role for efficient priming of tumour-specific T cells. In these T cells, P2X7R signalling is involved in orchestrating their migration, metabolic fitness, memory cell differentiation, and survival. Altogether, the demonstrated crucial role of P2X7R in initiation and maintenance of adaptive immune responses sends a clear message to approach P2X7R-inhibition in cancer with caution. While there is a strong rationale for inhibiting P2X7R in multiple cancers which exploit it to promote proliferation, invasion and metastasis, the conflicting outcomes of preclinical testing of P2X7R inhibitors in immunocompetent mouse models, and the fact that few P2X7R antagonists have made it to clinical testing in cancer patients, are further indications that more refined ways of targeting this pathway need to be devised.

Nevertheless, the selective expression of nfP2X7R on tumour constitutes an attractive target that could be exploited for cancer therapy. Expression of nfP2X7R variants by tumour cells provides an explanation for how they benefit from the proliferative advantages of P2X7R activation while simultaneously avoiding NICD and AICD. Indeed, recent pre-clinical and clinical studies of nfP2X7R- targeting antibodies and CAR-T cells have generated encouraging results (46, 47).

Emerging findings describing immune regulation by ARTs in inflammation and cancer are rewriting the script for how P2X7R-expression shapes the TME (116, 117). ARTs are potent triggers of NICD via P2X7R, particularly in NAD^+^-rich conditions such as the TME. Cytotoxic therapy directed at tumours may increase local levels of NAD^+^ and prime ART-mediated NICD in treated tumours. The recent discovery of how tumour-expressed ART1 allows tumours to co-opt the immune homeostatic mechanism of NICD suggests that targeted ART1-inhibition would counter this immune escape mechanism and maintain the viability of critical anti-tumour immune cells (55).

It is plausible that tumour-engaged T cells rely on the ATPase activity of CD39 and NADase activity of CD38 not only to temper their activation by generating adenosine precursors but also to avoid AICD and NICD induced by sustained exposure to high concentrations of ATP and NAD+ in solid tumours (118). In fact, Tregs and TRMs upregulate CD39 and CD38 in response to activation and TCR activation (116, 119). This cytoprotective role of ectonucleotidase expression may be especially crucial in the context of immunotherapy, where increased susceptibility to activation-induced apoptosis has been reported (120). Considering these findings, re-evaluation of clinical trial designs where ectonucleotidase inhibitors in combination with immunotherapy are warranted. Indeed, clinical testing of anti-CD38 antibodies daratumumab and isatuximab in combination with PD-1/PD-L1 inhibitors in patients with solid malignancies have generated negative survival outcomes, including early termination of one of the studies due to increased mortality in the combined treatment arm (121–123).

In summary, new insights have provided both cautionary tales and important clues for how the dysregulated P2X7R-signalling that occurs in tumours could be alternatively targeted to optimize immunotherapeutic treatments of cancer patients.

## Author contributions

RS: Writing – original draft, Writing – review & editing, Visualization. JR-Q: Writing – original draft, Writing – review & editing. MM: Writing – original draft, Writing – review & editing. BS: Writing – original draft, Writing – review & editing. EW: Writing – original draft, Writing – review & editing, Visualization.
